# Anatomical feasibility of an endovascular aortic arch repair with the NEXUS endograft in patients treated with a frozen elephant trunk procedure for aortic arch pathology

**DOI:** 10.1186/s42155-023-00355-0

**Published:** 2023-03-02

**Authors:** Ward Exelmans, Hozan Mufty, Geert Maleux, Peter Verbrugghe, Inge Fourneau

**Affiliations:** 1 Department of Vascular Surgery, University Hospitals Leuven, 3000 Leuven, Belgium; 2 Department of Radiology, University Hospitals Leuven, Leuven, Belgium; 3 Department of Cardiac Surgery, University Hospitals Leuven, Leuven, Belgium

**Keywords:** Aortic arch, Aortic stent graft, Endovascular repair, Anatomic feasibility

## Abstract

**Background:**

The aim of this study was to evaluate the feasibility of an endovascular repair, using the NEXUS™ Aortic Arch Stent Graft System, in a real-world cohort of patients, treated with a Frozen Elephant Trunk (FET) procedure for pathology involving the aortic arch.

**Results:**

The preoperative computed tomography angiography scans of 37 patients were retrospectively analyzed using a dedicated workstation. In total, seven patients (*N* = 7/37; 18.9%) were eligible for endovascular repair. This number increased to eleven patients (*N* = 11/37; 29.7%) if an additional relining of the distal aorta would be performed. Device suitability was 47.1% in patients (*N* = 8/17; 47.1%) with aortic arch aneurysm, 12.5% (*N* = 1/8; 12.5%) in patients with an acute Stanford type A dissection and 50% (*N* = 2/4; 50%) in patients with Crawford type II thoraco-abdominal aneurysm. The stent graft was not suitable for any of the two patients with chronic type B dissection (*N* = 0/2; 0%). In 22 patients (*N* = 22/37; 59.5%) an endovascular repair with this type of stent graft was not feasible due to an inadequate proximal sealing zone. There was no suitable brachiocephalic trunk landing zone in 13 patients (*N* = 13/37; 35.1%). There was no suitable distal landing zone distal in 14 patients (*N* = 14/37; 36.8%). This number decreased to ten patients (*N* = 10/37; 27.0%) when considering an additional relining of the distal aorta.

**Conclusions:**

Endovascular repair with the NEXUS single branch stent graft is feasible in a minority of this real-world cohort that underwent a Frozen Elephant Trunk procedure. However, the applicability of this device probably improves in cases with isolated aortic arch aneurysms.

## Background

Open surgery has been the gold standard to treat aortic arch pathologies for decades. Despite advances like the Frozen Elephant Trunk (FET) procedure this remains high risk surgery, requiring cardiopulmonary bypass and hypothermic circulatory arrest (Czerny et al. 2019; Tian et al. 2013). This results in at least 10–13% of patients being rejected for surgery due to their comorbidities (Pape et al. 2015). A less invasive endovascular approach might be a solution for those patients who are deemed unfit for open surgery. Over the last years a number of groups have reported their experience using branched endografts for aortic arch repair (Weijde et al. 2020; Ferrer et al. 2018; Tsilimparis et al. 2019; Spear et al. 2016). Custom-made multiple branch endografts have the advantage of being tailored to the specific anatomy of the patient but they have a delivery time of 4 – 8 weeks. This renders them unfit for use in urgent cases. The endovascular armamentarium is rapidly expanding, and off-the-shelf devices are upcoming. The NEXUS™ Aortic Arch Stent Graft System (Artivion Inc., Kennesaw, GA, USA) is a single outer branch off-the-shelf stent graft system developed to answer to the challenges of the anatomy of the aortic arch. Based on preoperative imaging from a cohort of patients treated for aortic arch pathology with a FET at our center, the present study evaluates the anatomic feasibility of an endovascular aortic arch repair using the NEXUS stent graft.

## Methods

### Device

**Fig. 1:**
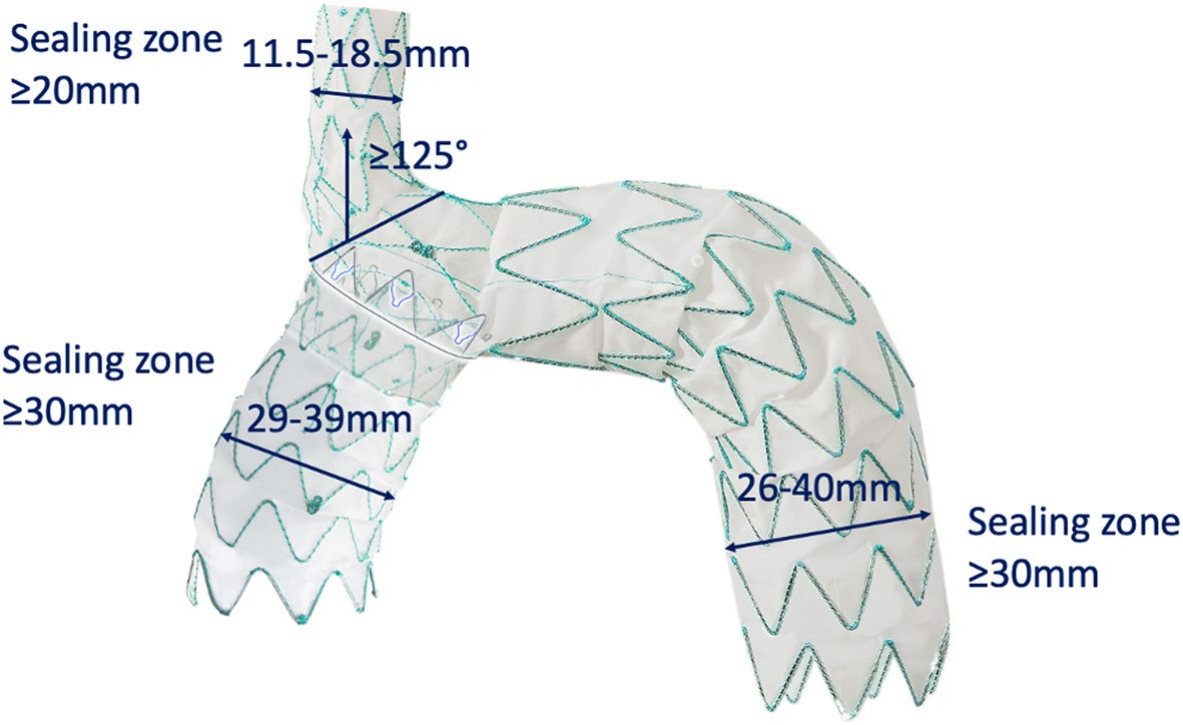
NEXUS™ Aortic Arch Stent Graft System with instructions for use

The NEXUS™ Aortic Arch Stent Graft System (NEXUS) is a CE approved off-the-shelf single branch stent graft system for aortic arch repair (Fig. 1). It is available in a 20Fr delivery system and consists of two different components. The main graft is deployed via a through-and-through wire from the brachiocephalic trunk to the descending thoracic aorta and has a docking sleeve facing the ascending thoracic aorta. The second stent graft is deployed in zone 0 and is connected to the main graft by a specially designed locking mechanism to ensure optimal sealing and fixation. Prior to endograft deployment a debranching of the supra-aortic vessels is mandatory. This means a right to left carotid-carotid cross-over bypass and a left carotid-subclavian bypass. The debranching can be performed in one procedure or staged.

### Patient cohort

All patients treated with a FET procedure for pathology involving the aortic arch at the department of Cardiac Surgery of the University Hospitals Leuven between June 2017 and May 2021, both in elective and urgent/emergent setting, were retrospectively reviewed. Baseline characteristics were collected from patient’s electronic medical records. Preoperative contrast enhanced computed tomography angiography (CTa) images were reviewed to assess the feasibility of a repair with the NEXUS endograft.

### Measurements

**Fig. 2:**
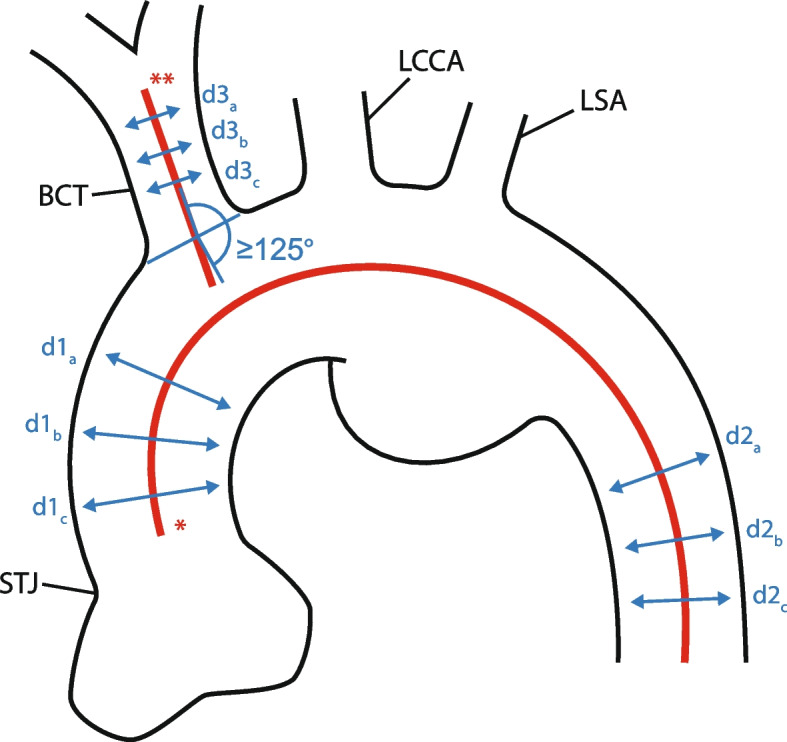
Schematic representations of the measurements performed on the CTa images. STJ: sinotubular junction BCT: brachiocephalic trunk, LCCA: left common carotid artery, LSA: left subclavian artery, *: center lumen line of the aorta, **: center lumen line of the brachiocephalic trunk, d1_a-c_: diameter measurements of the ascending aorta, d2_a-c_: diameter measurements of the descending aorta, d3_a-c_: diameter measurements of the brachiocephalic trunk

All images were uploaded to a dedicated workstation (S*yngo*.via, Siemens Healthcare GmbH, Erlangen, Germany). Feasibility was determined according to anatomic criteria derived from the instructions for use (IFU) (Fig. 1). A center lumen line was automatically created and manually adjusted based on multiplanar reconstructions. All diameter and length measurements were performed perpendicular to this center lumen line (Fig. 2). Maximum and minimum diameters were checked at different levels for each landing zone.

### Statistical analysis

Descriptive statistics were applied. Calculations were performed with Microsoft Excel Version 16.55 (Redmont, WA, USA).

## Results

Between June 2017 and May 2021, thirty-eight patients underwent a FET procedure. It was not possible to further analyze the CTa from one patient due to technical issues. Thirty-seven (*N* = 37/38; 97.4%) patients had a preoperative CTa that was eligible for further analysis. The mean age of these patients was 64.4 ± 2.1 years. Twenty-two (*N* = 22/37; 57%) patients were male. Other baseline characteristics are depicted in Table 1. The indications for intervention were aortic arch aneurysm (*N* = 17/37; 45.9%), acute Stanford type A dissection (*N* = 8/37; 21.6%), aneurysmal degeneration in chronic Stanford type B dissection (*N* = 7/37; 18.9%), Crawford type II thoraco-abdominal aneurysm (*N* = 4/37; 10.8%), non-A non-B dissection (*N* = 1/37; 2.7%). Twenty-eight patients (*N* = 28/37; 75.6%) were treated in elective circumstances. Nine patients (*N* = 9/37; 24%) were treated in emergency setting. In 13 (*N* = 13/37; 35.1%) patients a FET procedure alone was performed, in all other cases there was at least one associated procedure (Table 2).

**Table 1:** Baseline characteristics

Baseline characteristics (*N* = 37)	N (%) or Median (IQR)
Age	67 (57 – 73)
Gender	21 (57%) male
EuroSCORE II	5.58 (2.88 – 8.08)
Smoking	19 (51%)
Hypertension	25 (65%)
Dyslipidemia	21 (57%)
Diabetes mellitus	3 (8%)
COPD	5 (14%)
Dysrythmia	6 (16%)
Mild renal dysfunction (eGFR 60-89)	14 (38%)
Mild-moderate renal dysfunction (eGFR 45-59)	8 (22%)
Moderate renal dysfunction (eGFR 30-44)	4 (11%)
Previous cardiac surgery	12 (32%)
Previous aortic surgery	13 (35%)

*COPD* Chronic obstructive pulmonary disease

**Table 2:** Associated procedures

Procedure	N(%)
SCAR	9 (24%)
Aortic valve repair	7 (19%)
Bentall	7 (19%)
CABG	5 (14%)
Maze	1 (3%)
Pericardectomy	1 (3%)
Mitral valve plasty	1 (3%)

*SCAR* Supra-coronary aortic repair, *CABG* Coronary artery bypass graft

Zone 0 was inadequate as proximal landing zone in 22 patients (*N* = 22/37; 59.5%) (Table 3). In the majority of these patients (*N* = 18/22; 81.8%%) zone 0 was too wide (> 39 mm) for the NEXUS endograft. In four other patients (*N* = 4/22; 18.2%) the proximal sealing zone was too short (< 30 mm). An inadequate landing zone at the level of the brachiocephalic trunk (BCT) excluded 13 patients (*N* = 13/37; 35.1%). The BCT was too wide (> 18.5 mm) in three patients (*N* = 3/37; 8.1%). The sealing zone was less than 20 mm in seven patients (*N* = 7/37; 18.9%). In four patients (*N* = 4/37; 10.8%) the take-off angle between the BCT and the aortic arch was less than 125°.

**Table 3:** Feasibility based on diameter, sealing zone and angle measurements

Aortic arch feasibility (*N* = 37)	N(%)
Proximal landing zone 29–39 mm	19 (51%)
Proximal sealing zone ≥ 30 mm	15 (41%)
Distal landing zone 26–40 mm	25 (68%)
Distal sealing zone ≥ 30 mm	23 (68%)
BCT 11.5–18.5 mm	34 (92%)
BCT sealing zone ≥ 20 mm	30 (81%)
Take of angle BCT^a^ ≥ 125°	33 (89%)
Overall feasibility	7 (19%)
Access feasibility (*N* = 29)	
Adequate diameter of the iliac and femoral arteries	28 (97%)

*BCT* Brachiocephalic trunk

^a^ angle between the brachiocephalic trunk and the aortic arch perpendicular

An inadequate distal landing zone excluded 13 patients (*N* = 13/37; 35.1%). The distal landing zone was too wide (> 40 mm) in 12 patients (*N* = 12/13; 92.3%). In one other patient (*N* = 1/13; 7.7%) the distal sealing zone was less than 30 mm long. Considering a relining of the distal aorta, the number of excluded patients decreased to nine (*N* = 9/37; 24.3%).

Combining all these limitations, aortic arch repair with the NEXUS endograft was anatomically feasible in seven patients (*N* = 7/37; 18.9%). Additional relining of the distal aorta would raise this to eleven patients (*N* = 11/37; 29.7%). Looking at the underlying disease, endovascular repair was possible in eight patients with an aortic arch aneurysm (*N* = 8/17; 47.1%), one patient with an acute Stanford type A dissection (*N* = 1/8; 12.5%), two patients with a Crawford type II thoraco-abdominal aortic aneurysm (*N* = 2/4; 50%). None of the patients with aneurysmal degeneration in chronic Stanford type B dissection were suitable for endovascular treatment, nor was one patient with a non-A non-B dissection.

In 29 cases (*N* = 29/37; 78.4%) imaging extended as low as the iliac and femoral arteries to allow for access evaluation. Bilateral access was feasible in 25 patients (*N* = 25/37; 66.8%). In one female patient (*N* = 1/37; 2.7%) the femoral and iliac vessels were too narrow to allow the delivery device to pass through. In three patients (*N* = 3/37; 8.1%) access was only possible via the left iliac artery due to an occlusion of the right iliac artery.

## Discussion

Endovascular repair for aortic arch pathology is one of the last hurdles toward a full endovascular treatment of the aorta. In the present study, the feasibility of the NEXUS stent graft system was assessed in patients who underwent a FET procedure in our center. In 29.7% of patients an endovascular repair would have been anatomically feasible using the NEXUS stent graft with/without additional endografts for distal seal. Smorenburg et al. reported a feasibility of the NEXUS stent graft in 20.3% (*N* = 31/153) of cases. Considering the associated procedures performed, the effective feasibility in the present study is reduced to seven (*N* = 7/37; 18.9%). Whether or not these associated procedures could be avoided is uncertain. Moreover, for some of these procedures, a less invasive alternative is available e.g., a percutaneous coronary intervention instead of coronary artery bypass grafting or transcatheter aortic valve replacement instead of a surgical approach. These options must be evaluated on a case-to-case basis.

There is currently one competitor with a comparable off-the-shelf outer branch design available: TAG Thoracic Branch Endograft (W.L. Gore & Associates, Flagstaff, AZ, USA). The reported feasibility of the TAG Thoracic Branch Endograft was 30.1% (Smorenburg et al. 2020).

An ascending aorta that was too wide was the main reason for exclusion in the present study (48.6%). This is in line with the findings of other feasibility studies (range of exclusion for the off-the-shelf devices varied from 13.7–65%) (Smorenburg et al. 2020; Fujimura et al. 2017). The diameter of the ascending aorta should be within the range of 29-39 mm for the NEXUS device with the largest device of the ascending module measuring 43 mm. A stent graft diameter of ≥ 42 mm is a risk factor for retrograde type A dissection (Kudo et al. 2022), which is a potentially fatal complication. Creating a surgical graft-landing zone can solve this problem in patients with a wide ascending aorta and makes this patient group more suitable for endo-arch repair (Verscheure et al. 2019). Clinical experience with the NEXUS graft is limited. Planer et al. recently reported on the initial clinical experience (Planer et al. 2021). No retrograde dissections were reported, no further specifications on device diameter were provided. Recently, 3-year outcome results were published. No device related deaths were observed during follow-up. Cumulative incidence of unplanned device or procedure related interventions at 3 years was 29% (D’Onofrio et al. 2022).

Looking at pathological features, implantation of the NEXUS stent graft was feasible in 35.3% of patients with thoracic aneurysm compared to 20.3% reported by Smorenburg et al. This marked difference might be a consequence of a selection bias in the present cohort since all patients were eligible for surgical repair. An overview of feasibility of different devices in patients with a Stanford type A dissection can be found in Table 4. Notably, feasibility rates of the TAG Thoracic Branch Endograft are markedly higher in the study from Fujimura et al. compared to others feasibility studies (Smorenburg et al. 2020; Fujimura et al. 2017). This discrepancy can possibly be explained by the anatomical variability between Caucasian and Asian patient groups (Fujimura et al. 2017; Cheng et al. 2004). Secondly, only patients with an acute type A aortic dissection were included in the study of Fujimura et al. (Fujimura et al. 2017).

**Table 4:** Feasibility of stent graft implantation in patients with Stanford type A dissection

	Current study *N* = 8	Smorenburg et al. *N* = 80	Fujimura et al. *N* = 131
NEXUS Stent Graft System, Artivion Inc., Kennesaw, GA, USA	1/8 (12.5%)	8/80 (10%)	-
TAG Thoracic Branch Endograft W.L. Gore & Associates Flagstaff, AZ, USA	-	6/80 (7.5%)	60/131 (45.8%)
Zenith Arch Branched Device Cook Medica, Bloomington, IN, USA	-	2/80 (2.5%)	9/131(6.9%)

One of the difficulties in aortic arch repairs are neurologic complications. In the present cohort there was a stroke rate of 5 and 3% spinal cord ischemia after treatment with a FET. No perioperative death occurred within thirty days after surgery. The NEXUS stent graft system is specifically developed to reduce neurologic risks. Recent results from the implantation of the NEXUS endograft in 28 patients demonstrated a thirty-day mortality of 7.1% (Planer et al. 2021). Cerebro-vascular complications and spinal cord ischemia were reported to be 3.6 and 0% respectively. These are promising results and comparable to the experience of Verscheure et al., they described the implantation of the multi-branch Zenith Arch branch graft in 70 patients with a thirty-day mortality and cerebrovascular complication rate of 2.9 and 2.9%, respectively(Verscheure et al. 2019). Spinal cord ischemia was seen in none of the patients. The mortality rate after FET procedure is very low. Possible explanations are firstly, that patients included for an endovascular repair are more fragile, with an increased perioperative risk and secondly, experience with aortic arch endograft is limited compared to open cardiac surgery. Looking at cerebro-vascular complications, there is a low incidence the NEXUS group which might be explained by reduced endovascular manipulation of the supra-aortic vessels. However, the additional risk of debranching should be considered when interpreting data from the NEXUS endograft. What is more, a debranching of the supra-aortic vessels prior to deployment might be challenging in acute settings like interventions for Stanford type A dissection. Surgical alternatives like the periscope technique can be used to circumnavigate this problem. However, this technique is currently not recommended and falls outside the IFU (Czerny et al. 2019; Anwar and Hamady 2020).

The main limitation of this study is the selection bias. We only evaluated patients that passed the preoperative screening and were found fit for surgery. Since open repair of the aortic arch is regarded as high-risk surgery, a substantial number of patients was probably rejected for surgery (Czerny et al. 2019; Pape et al. 2015). These are the patients that potentially benefit the most from a less invasive endovascular approach.

## Conclusion

Endovascular arch repair is gaining more interest over the last years. Next to custom made devices, off-the-shelf endografts are upcoming. Endovascular repair with the NEXUS single branch stent graft is feasible in a minority of this real-world cohort that underwent a FET procedure. However, the applicability of this device probably improves in cases with isolated aortic arch aneurysms.

## Data Availability

The datasets used and/or analysed during the current study are available from the corresponding author on reasonable request.

## Abbreviations

BCT: Brachio-cephalic trunk
CTa: Computed tomography angiography
FET: Frozen elephant trunk
IFU: Instruction for use

## References

[CR1] Anwar MA, Hamady M (2020). Various Endoluminal Approaches Available for Treating Pathologies of the Aortic Arch. CardioVascIntervent Radiol..

[CR2] Cheng SW, Ting AC, Ho P, Poon JT (2004). Aortic aneurysm morphology in Asians: features affecting stent-graft application and design. Endovasc Ther.

[CR3] Czerny M, Schmidli J, Adler S, van den Berg JC, Bertoglio L, Carrel T (2019). Editor’s Choice – Current Options and Recommendations for the Treatment of Thoracic Aortic Pathologies Involving the Aortic Arch: An Expert Consensus Document of the European Association for Cardio-Thoracic Surgery (EACTS) & the European Society for Vascular Surgery (ESVS). Eur J Vasc Endovasc Surg.

[CR4] D’Onofrio A, Lachat M, Mangialardi N, Antonello M, Schelzig H, Chaykovska L (2022). Three-year follow-up of aortic arch endovascular stent grafting with the Nexus device: results from a prospective multicentre study. Eur J Cardiothorac Surg.

[CR5] Ferrer C, Coscarella C, Cao P (2018). Endovascular repair of aortic arch disease with double inner branched thoracic stent graft: the Bolton perspective. J Cardiovasc Surg (torino).

[CR6] Fujimura N, Kawaguchi S, Obara H, Yoshitake A, Inoue M, Otsubo S (2017). Anatomic feasibility of next-generation stent grafts for the management of type a aortic dissection in Japanese patients. Circ J.

[CR7] Kudo T, Kuratani T, Shirakawa Y, Shimamura K, Kin K, Sakamoto T (2022). Effectiveness of Proximal Landing Zones 0, 1, and 2 Hybrid Thoracic Endovascular Aortic Repair: A Single Centre 12 Year Experience. Eur J Vasc Endovasc Surg.

[CR8] Pape LA, Awais M, Woznicki EM, Suzuki T, Trimarchi S, Evangelista A (2015). Presentation, diagnosis, and outcomes of acute aortic dissection: 17-year trends from the international registry of acute aortic dissection. J Am Coll Cardiol.

[CR9] Planer D, Elbaz-Greener G, Mangialardi N, Lindsay T, Schelzig H, Chaykovska L (2021). NEXUS^TM^ Arch: a multicenter study evaluating the initial experience with a novel Aortic Arch Stent Graft System.

[CR10] Smorenburg SPM, Montesano M, Hoogteijling TJ, Truijers M, Symersky P, Jansen EK (2020). Anatomic suitability for branched thoracic endovascular repair in patients with aortic arch pathological features. J Am Heart Assoc.

[CR11] Spear R, Haulon S, Ohki T, Tsilimparis N, Kanaoka Y, Milne CPE, et al. Editor’s choice - Subsequent results for arch aneurysm repair with inner branched endografts. Eur J Vasc Endovasc Surg. 2016;51(3):380-5. 10.1016/j.ejvs.2015.12.002 26818022

[CR12] Tian DH, Wan B, Bannon PG, Misfeld M, LeMaire SA, Kazui T (2013). A meta-analysis of deep hypothermic circulatory arrest versus moderate hypothermic circulatory arrest with selective antegrade cerebral perfusion. Ann Cardiothorac Surg.

[CR13] Tsilimparis N, Detter C, Law Y, Rohlffs F, Heidemann F, Brickwedel J (2019). Single-center experience with an inner branched arch endograft. J Vasc Surg.

[CR14] van der Weijde E, Heijmen RH, van Schaik PM, Hazenberg CEVB, van Herwaarden JA (2020). Total Endovascular Repair of the Aortic Arch: Initial Experience in the Netherlands. Ann Thorac Surg.

[CR15] Verscheure D, Haulon S, Tsilimparis N, Resch T, Wanhainen A, Mani K, et al. Endovascular treatment of post type A chronic aortic arch dissection with a branched endograft: early results from a retrospective International multicenter study. Ann Surg. 2021;273(5):997-1003. 10.1097/SLA.0000000000003310 30973389

